# A randomised, multi-centre, prospective, observer and patient blind study to evaluate a non-absorbable polypropylene mesh vs. a partly absorbable mesh in incisional hernia repair

**DOI:** 10.1007/s00423-012-1009-6

**Published:** 2012-10-03

**Authors:** A. Rickert, P. Kienle, A. Kuthe, P. Baumann, R. Engemann, J. Kuhlgatz, M. von Frankenberg, H. P. Knaebel, M. W. Büchler

**Affiliations:** 1 Department of Surgery, University Medical Centre Mannheim, Mannheim, Germany; 2 Department of Surgery, DRK Hospital Clementinenhaus, Hannover, Germany; 3 Department Medical Scientific Affairs, Aesculap AG, Tuttlingen, Germany; 4 Surgical Clinic I, Clinical Centre Aschaffenburg, Aschaffenburg, Germany; 5 Department of Surgery, Helios Clinic, Northeim, Germany; 6 Department of Surgery, Hospital of Salem, Heidelberg, Germany; 7 Department of Surgery, University of Heidelberg, Heidelberg, Germany

**Keywords:** Incisional hernia, Lightweight mesh, Patient-reported outcome, Quality of life

## Abstract

**Background:**

The implantation of a polymer mesh is considered as the standard treatment for incisional hernia. It leads to lower recurrence rates compared to suture techniques without mesh implantation; however, there are also some drawbacks to mesh repair. The operation is more complex and peri-operative infectious complications are increased. Yet it is not clear to what extent a mesh implantation influences quality of life or leads to chronic pain or discomfort. The influence of the material, textile structure and size of the mesh remain unclear. The aim of this study was to evaluate if a non-absorbable, large pore-sized, lightweight polypropylene (PP) mesh leads to a better health outcome compared to a partly absorbable mesh.

**Methods/design:**

In this randomised, double-blinded study, 80 patients with incisional hernia after a median laparotomy received in sublay technique either a non-absorbable mesh (Optilene® Mesh Elastic) or a partly absorbable mesh (Ultrapro® Mesh). Primary endpoint was the physical health score from the SF-36 questionnaire 21 days post-operatively. Secondary variables were patients' daily activity score, pain score, wound assessment and post-surgical complications until 6 months post-operatively.

**Results:**

SF-36, daily activity and pain scores were similar in both groups after 21 days and 6 months, respectively. No hernia recurrence was observed during the observation period. Post-operative complication rates also showed no difference between the groups.

**Conclusion:**

The implantation of a non-absorbable, large pore-sized, lightweight PP mesh for incisional hernia leads to similar patient-related outcome parameters, recurrence and complication rates as a partly absorbable mesh.

## Background

### Rationale

Despite of all efforts to find an optimised abdominal fascia closure after laparotomy that minimises the risk for incisional hernia, the incidence of this complication is still as high as 20 % [1, 2]. Incisional hernias enlarge over time which makes repair more difficult. Moreover, serious complications such as bowel obstruction, incarceration and strangulation or entero-cutaneous fistula may occur over time. Therefore, elective hernia repair is indicated to avoid these complications and potential consecutive emergency surgery with a worse outcome [3]. From the perspective of the patient, other reasons for hernia repair may have a higher priority: everyday clinical practice shows that most patients with incisional hernia are attended to by the surgeon because their hernia causes pain, discomfort or limitations in daily life [4].

The ideal hernia repair procedure should combine a minimal recurrence and complication rate with a maximal reduction of pain and improvement of quality of life. Currently, no laparotomy closure can avoid incisional hernias. The INLINE systematic review showed the lowest incisional hernia rate after primary laparotomy closure using a continuous slowly absorbable suture in an elective setting [5].

As recurrences rates after suture repair are as high as 58 % [4, 6] the implantation of a prosthetic mesh is nowadays considered as the standard treatment. Recurrence rates between 2 % and 17 % are reported for mesh repair, depending on whether the mesh is placed in a sublay or onlay position [7, 8]. The lower recurrence rate in mesh repair is somewhat flawed by a slightly increased wound infection rate [9].

Several mesh types are available which differ in material, textile structure, pore size, weight, elasticity, tissue reaction, biocompatibility and absorption time. Most surgeons use large pore-sized, lightweight meshes which show an improved integration of the mesh in the abdominal wall tissue and reduced foreign body reaction compared to heavyweight meshes [10]. A clinically relevant difference in pain and patient discomfort was not found between both types of meshes [11, 12]. Lightweight meshes are also available with an absorbable component, which are thought to produce a reduced body reaction to the foreign mesh and to improve integration into the surrounding tissue leading to an increase tensile strength in the first weeks after implantation.

### Purpose

The aim of this study was to assess health-related quality of life of patients having undergone sublay incisional hernia repair with either a non-absorbable or a partly absorbable mesh. Few prospective studies have evaluated the influence of the mesh structure and other features on the quality of life after incisional hernia repair. Until now, no randomised controlled trials have addressed this particular topic.

## Methods/design

### Study support

The study protocol was published to ensure transparency of the trial [13]. The responsible ethic committee approved the study project. The trial was initiated and sponsored by Aesculap AG, Tuttlingen, Germany and conducted in cooperation with the contract research organisation (CRO) Dr. Med Lenhard & Partner GmbH, Overath, Germany. The CRO was responsible for monitoring, biostatistics and database maintenance. Aesculap AG was responsible for the project management and all trial-related meetings. The study was registered by Aesculap AG under www.clinicaltrials.gov.

### Study design and study population

The study was designed as a prospective, randomised, double-blinded multicentre study. Six hospitals throughout Germany participated in the study (DRK Hospital Clementinenhaus, Hannover; Helios Clinic, Northeim; Clinical Centre Aschaffenburg; University of Heidelberg; Hospital Salem, Heidelberg; and University Clinical Centre Mannheim).

The inclusion and exclusion criteria as well as the surgical intervention have been described in the study protocol [13]. Eighty patients with an elective incisional hernia repair in sublay technique were included. Only patients over 18 years with vertical aponeurotic incision and an incisional hernia size of 3 cm or more were suitable for the study. Exclusion criteria were previous mesh repair at the same site, acute incarcerated hernia, additional surgical treatment at the same time and anticoagulation therapy. Written informed consent was obtained from all participating patients.

### Surgical procedure

To assure comparability and minimise potential bias, the operation procedure and the peri-operative treatment were strictly standardised. The operation was initiated with a vertical median incision. After classification of the hernia according to the Schumpelick criteria [14], a space was created between both posterior sheaths and the rectus muscles. The posterior fascia was closed with a running non-absorbable suture (polypropylene, PP). The mesh was placed in a sublay position between the rectus muscles and the posterior rectus sheath or the peritoneum inferior of the arcuate line, respectively. The mesh was fixed by single knots every 3 cm using a monofilament, non-absorbable suture (PP) with an overlap of the abdominal wall defect of 5 cm in each direction. The midline was closed with a continuous running monofilament non-absorbable suture with a 4:1 ratio between suture length and incision length. Two Redon drains were placed to the mesh. The skin was closed with tacks. Food intake started on day one post-operatively with light food.

### Meshes

We compared the Optilene® Mesh Elastic (30 × 30 cm) manufactured by B. Braun Aesculap with the Ultrapro® Mesh (30 × 30 cm) by Johnson & Johnson. The two meshes have large pores based on PP. Optilene® Mesh Elastic is made of pure PP and is not absorbable (weight 48 g/m^2^, pore size 2.9–3.2 mm). The Ultrapro® Mesh is a partly absorbable mesh (PP plus polyglecaprone, PG (PP-PG) (~1:1), weight 65 g/m^2^, after absorption of PG weight 28 g/m^2^, pore size 1.9–2.2 mm).

### Study objectives

The primary objective was the comparison of the physical health score of the SF-36 questionnaire 21 days after implantation of either the Optilene® Mesh Elastic (PP) or the Ultrapro® Mesh (PP-PG). Secondary objectives were patients' daily activity, pain scores and specific post-operative complications, especially wound-related problems such as infection or seroma and the hernia recurrence rate until 6 months post-operatively.

### Follow-up, SF-36 score

The follow-up for each patient was 6 months. Patients were seen for clinical follow-up at the day of discharge as well as 21 days and 6 months after surgery, respectively. A phone call was conducted at 4 months. Subjective variables were assessed by the SF-36 questionnaire, a daily activity questionnaire and a pain score (scale from 0 = no pain to 5 = maximum pain).

The SF-36 score was used as the best known and validated score in measuring physical and mental health [15]. It consists of 36 items which are grouped under eight major health quality domains: physical function, role limitations due to physical function, bodily pain and general health are summarised to a physical health score; vitality, social function, and role limitations due to emotional function and mental health add up to the mental health score. The scale ranges from 0 to 100, where 100 describes best health status. The daily activity questionnaire consisted of 11 questions regarding every day activities (i.e., getting up, heavy lifting and defecation) and the limitations experienced by the patient (no, light, medium, and strong limitation).

On the first visit after inclusion of the patient, the following data were collected: demographic data, determination of risk factors, medical history, history of the incisional hernia to be repaired and concomitant medication. A clinical examination was performed and a general health status was obtained. The hernia was classified according to the Schumpelick classification [14]:

- Class I Hernia defect <2 cm, barely visible whilst standing and lying, detectable by sonography or palpation
- Class II Hernia defect <4 cm, visible as protrusion whilst standing, flat when lying resp. spontaneously reducible
- Class IIb Hernia defect <4 cm, in addition not reducible by taxis
- Class III Hernia defect >4 cm, during standing visible as a bulge, whilst lying flat and spontaneously reducible
- Class IV Hernia defect >4 cm, during standing and lying visible, reposition not spontaneously
- Class IVb Hernia defect >4 cm, in addition not reducible by taxis
- Class V total defect of the abdominal wall.

SF-36 questionnaires, daily activity questionnaires and pain scores were recorded pre-operatively, 21 days and 6 months after surgery, respectively. The pain score was additionally documented on the day of discharge.

Wound assessment was performed on day of discharge, 21 days and 6 months after surgery. Variables recorded were bleeding, hernia recurrence, wound infection/haematoma, seroma formation and chronic pain. If clinically indicated, an ultrasound was done. Adverse events (AEs) and serious AEs were documented by the investigator until 6 months post-operatively.

### Randomisation and blinding

A randomisation list using a block size of four was generated by the statistician. Sealed opaque numbered envelopes with a balanced distribution of the meshes were prepared according to the randomisation list. Patients who matched inclusion criteria were randomised by opening sealed opaque envelopes containing the mesh to be implanted. The envelopes were opened by the surgeon in the operation room. The investigator who conducted the follow-up examinations as well as the patient had no access to the document showing which mesh was used. Therefore, at least two different persons per centre were involved in the study, one who performed the surgery and one who conducted the follow-up examinations. Together with the meshes, each trial centre received emergency envelopes with the information of treatment allocation.

### Statistics

Patients were analysed using the intention to treat (ITT) principle. A patient belonged to the ITT population after confirmed completion of the surgical procedure by an enrolment fax. The primary endpoint was the change of the SF-36 physical health score between baseline and 21 days after surgery. Significance levels were set at *p* < 0.05 (two-sided). Due to the lack of any empirical data for the primary endpoint in the population under investigation, there was substantial uncertainty with respect to overall rate and treatment to be expected. As a consequence, the assumptions to be made for sample size calculation were highly uncertain. Therefore, the study was performed with 80 patients, a sample size which could be recruited multi-centric in an appropriate period. For comparison of the treatment groups, Wilcoxon rank-sum tests were calculated, of which only the test of the primary endpoint has confirmative character. Secondary endpoints and safety assessment including AEs and serious AEs until 6 months post-operatively were analysed descriptively. Thus, no Bonferroni correction of the *p* values was done. The analysis was performed after closing the database by using SAS system 9.2 (SAS Inc.; Cary, NC, USA) according to the previously specified statistical analysis plan. An interim analysis was not performed.

## Results

### Patients and hernia characteristics

**Fig. 1:**
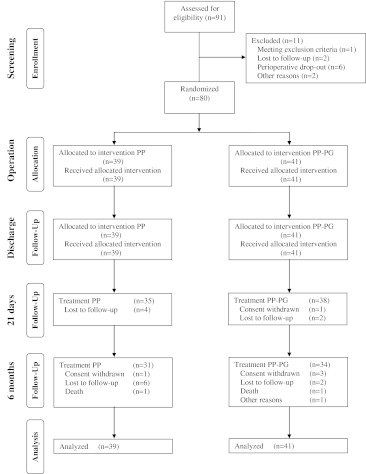
CONSORT flow chart

Between July 2006 and March 2010, 91 patients were screened for study inclusion. Eleven patients were excluded for different reasons; 80 patients were randomised and included in the study. The follow-up of the last patient was completed in October 2010. Thirty-nine patients received the non-absorbable PP mesh, and in 41 patients, the partly absorbable PP-PG mesh was implanted. All 80 patients were analysed; 31 patients in the PP mesh group and 34 patients in the PP-PG mesh group completed the study as planned. After 21 days, the primary endpoint was reached by 35 patients in the PP mesh group and 38 patients in the PP-PG mesh group, respectively (Fig. 1).

The two treatment groups were comparable regarding age distribution and body mass index (BMI). To assess the patients' degree of everyday activity, they were asked about their employment status, type of work and type of leisure activity. Employment status was similar in both groups. Whilst in the PP mesh group, more patients judged their work as largely sedentary (25.64 % vs. 12.2 %), more patients in the PP-PG mesh group had an active work (24.4 % vs. 12.82 %). Most patients in the two groups were moderately active or very active in their leisure time, with a trend to more active patients in the PP-PG mesh group (Table 1).

**Table 1:** Demographic data and medical history

	PP mesh group (*N* = 39)	PP-PG mesh group (*N* = 41)
Age (years), mean ± SD	63.26 ± 9.54	61.65 ± 12.2
BMI (kg/m^2^), mean ± SD		
Visit 1	28.71 ± 4.79	27.48 ± 4.57
Visit 6	28.86 ± 5.05	28.53 ± 4.81
Ethnic groups		
Caucasian	39 (100 %)	39 (95.12 %)
Black	0 (0 %)	1 (2.44 %)
Other	0 (0 %)	1 (2.44 %)
Employment status		
Full-time	10 (25.64 %)	13 (31.71 %)
Part-time	4 (10.26 %)	3 (7.32 %)
Self-employed	2 (5.13 %)	1 (2.44 %)
Retired	18 (46.15 %)	20 (48.78 %)
Unemployed	5 (12.82 %)	4 (9.76 %)
Occupational type		
Largely sedentary	10 (25.64 %)	5 (12.2 %)
Predominantly sedentary	5 (12.82 %)	4 (9.76 %)
Active work	5 (12.82 %)	10 (24.4 %)
Essentially always on feet	1 (2.56 %)	5 (12.2 %)
Very labour intensive	2 (5.13 %)	4 (9.76 %)
Not working	16 (41.03 %)	13 (31.71 %)
Type of activity		
Largely sedentary	3 (7.69 %)	3 (7.32 %)
Fairly sedentary	4 (10.26 %)	8 (19.51 %)
Moderately active	24 (61.54 %)	20 (48.78 %)
Very active	7 (17.95 %)	10 (24.39 %)
Always on feet	1 (2.56 %)	0 (0 %)
ASA classification		
1	3 (7.69 %)	3 (7.32 %)
2	24 (61.54 %)	19 (46.34 %)
3	10 (25.64 %)	19 (46.34 %)
4	2 (5.13 %)	0 (0 %)
Co-morbidities		
Diabetes	6 (15.38 %)	6 (14.63 %)
Chronic smoker	9 (23.08 %)	10 (24.39 %)
COAD	5 (12.82 %)	5 (12.2 %)
Chronic bronchitis	3 (7.69 %)	6 (14.63 %)
Renal insufficiency	0 (0 %)	4 (9.76 %)
Malnutrition	0 (0 %)	0 (0 %)
Corticoid therapy	1 (2.56 %)	1 (2.44 %)
Obesity	9 (23.08 %)	11 (26.83 %)
Chronic constipation	2 (5.13 %)	4 (10.0 %)
Abdominal aneurysm	0 (0 %)	1 (2.44 %)

*COAD* chronic obstructive airway disease, *ASA* American Society of Anesthesiologists, *BMI* body mass index, *SD* standard deviation, *PP* polypropylene, *PP-PG* polypropylene plus polyglecaprone

Medical history showed that patients in both groups had the same co-morbidities and risk factors, except renal insufficiency (PP 0 % vs. PP-PG 9.76 %). More patients classified ASA3 or ASA4 were found in the PP-PG group (46.3 % vs. 30.77 % in the PP group).

The type of operation after which the hernia developed is shown in Table 2. The incisional hernias in this study occurred mostly after colorectal resections (33.3 % PP vs. 36.6 % PP-PG). The hernia developed at a mean of 1.6 years after the previous operation in the PP-PG mesh group and 2 years in the PP-mesh group. Over 70 % of patients in both groups reported that the hernia was always present. Between 75 and 91 % in both groups reported that the hernia was predominant during laughing, coughing, defecation and heavy lifting. The size of the hernia tended to be higher in the PP-PG mesh-group, with over 85 % of patients having a hernia classified grade III or higher vs. 70 % in the PP-mesh group.

**Table 2:** Hernia characteristics

	PP mesh group (*N* = 39)	PP-PG mesh group (*N* = 41)
Type of initial surgery		
Upper GI surgery	4 (10.26 %)	7 (17.07 %)
Aortic surgery	3 (7.69 %)	4 (9.76 %)
Hernia surgery	4 (10.26 %)	4 (9.76 %)
Expl. laparotomy	3 (7.69 %)	1 (2.44 %)
Obstetric surgery	1 (2.56 %)	4 (9.76 %)
Colorectal surgery	13 (33.33 %)	15 (36.59 %)
Small intestine	1 (2.56 %)	1 (2.44 %)
Other laparotomy	6 (15.38 %)	1 (2.44 %)
Other surgery	2 (5.13 %)	3 (5.13 %)
Unknown	2 (5.13 %)	1 (2.44 %)
Hernia classification^a^		
II	11 (28.21 %)	4 (9.76 %)
IIb	2 (5.13 %)	1(2.44 %)
III	16 (41.03 %)	21 (51.22 %)
IV	6 (15.38 %)	13 (31.71 %)
IVb	4 (10.26 %)	1 (2.44 %)
V	0 (0 %)	1 (2.44 %)
Development of hernia (years), mean ± SD	2.07 ± 3.84	1.62 ± 3.18

*SD* standard deviation, *PP* polypropylene, *PP-PG* polypropylene plus polyglecaprone

^a^Schumpelick classification

### Post-operative complications and adverse events

The complication rate was similar in both groups. Whilst in the PP-PG group, more seroma formations were observed after 21 days (21.6 % vs. 17.14 %), more patients in the PP group had a minor haematoma at 21 days (25.71 % vs. 16.22 %). After 6 months, 6.45 % in the PP-group and 2.94 % in the PP-PG group still had a seroma. One patient needed surgery for a major wound infection after the implantation of a PP-PG mesh. Three patients had a post-operative haematoma that required surgery (two with partly absorbable mesh vs. one with non-absorbable mesh). No hernia recurrence occurred during the follow-up period.

### SF-36 score

**Fig. 2:**
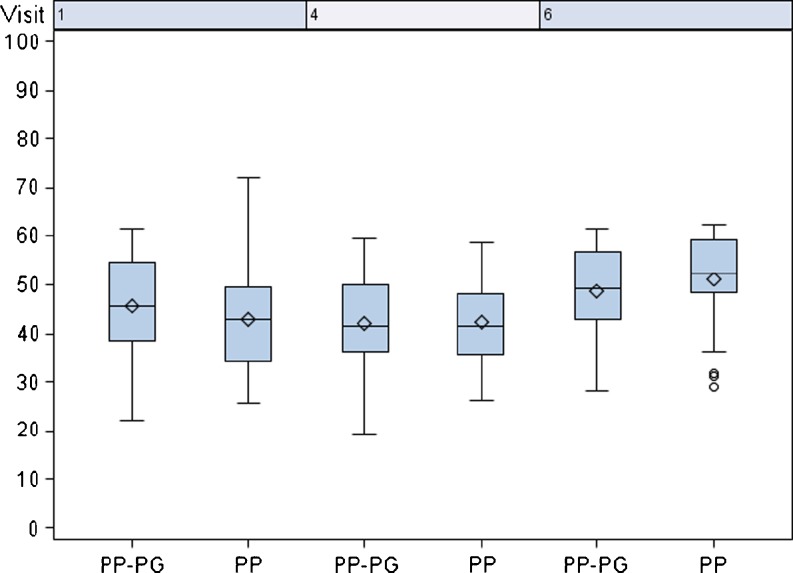
Standardized physical health score. Visit 1: pre-op; visit 4: 21 days postoperatively (primary endpoint), visit 6: 6 months post-operatively

There was no difference in the SF-36 physical health score between both mesh groups after 21 days, with a mean value of 41.95 for the PP-PG mesh vs. 42.46 for the PP mesh. After 6 months, the score increased in both groups to 48.75 and 51.16, respectively. The increase in the PP group was slightly higher than in the PP-PG group (Fig. 2).

**Fig. 3:**
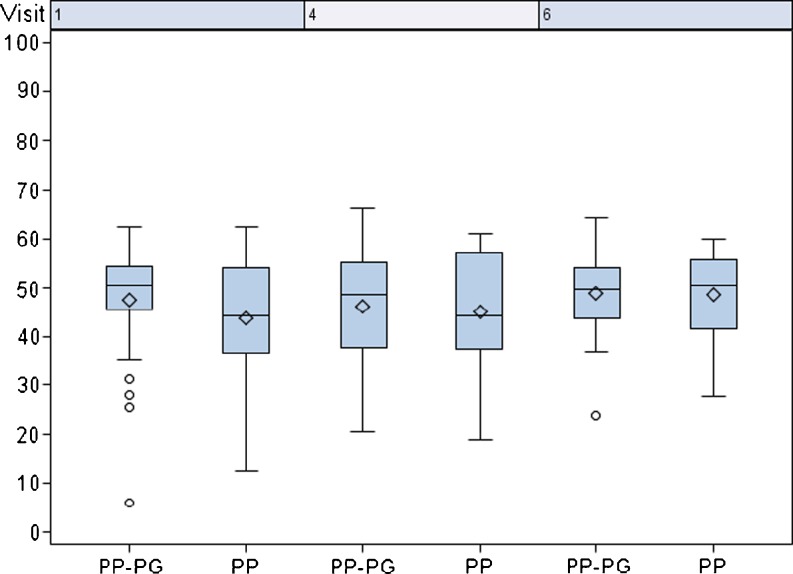
Standardized mental health score. Visit 1: pre-op; visit 4: 21 days post-operatively, visit 6: 6 months post-operatively

**Fig. 4:**
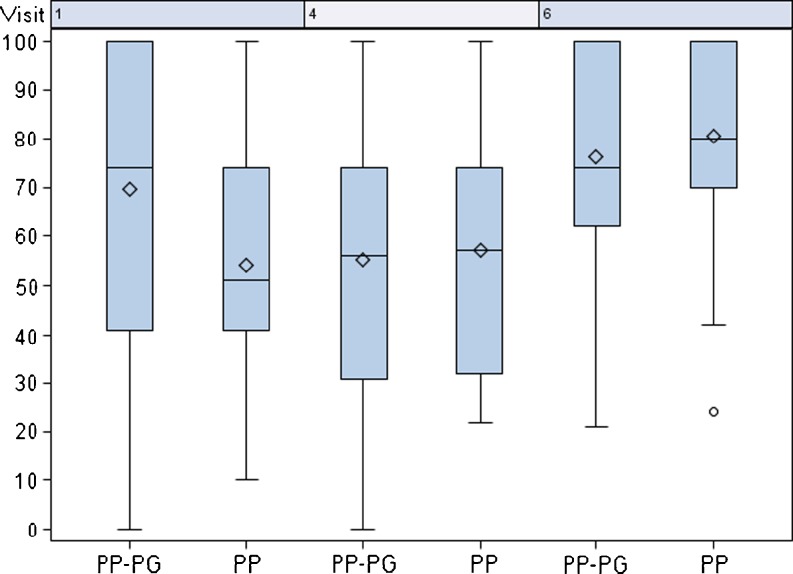
SF 36-item bodily pain. Visit 1: pre-op; visit 4: 21 days post-operatively, visit 6: 6 months post-operatively

The values of the eight domains of the SF-36 health survey obtained for each mesh group are shown in Table 3. A summary of the mental health components are demonstrated in Fig. 3. For the SF-36 items Role-Emotional (*p* = 0.033) and Bodily Pain (*p* = 0.015), a significant lower score was found at baseline in the PP mesh group compared to the PP-PG mesh group. In contrast, an increase in the SF-36 item Role-Emotional was seen from pre-operatively until 6 months post-operatively in the PP mesh group from 58.33 to 78.33 vs. 75.22 to 79.05 in the PP-PG mesh group (data not shown). The difference in the score for the item bodily pain was significantly higher from baseline to 21 days (*p* = 0.04) from baseline to 6 months (*p* = 0.036) in the PP mesh group in comparison to the PP-PG mesh group (Fig. 4). A slight significant difference was observed for the item Role-Physical for patients receiving a PP mesh until 21 days post-operatively (*p* = 0.049), but after 6 months, no difference was seen between the two mesh groups (data not shown).

**Table 3:** SF-36 health survey

	PP mesh		PP-PG mesh	
	21 days post-op	6 months post-op	21 days post-op	6 months post-op
Physical functioning	61.01 ± 25.35	80.16 ± 23.67	64.86 ± 23.65	77.24 ± 20.77
Role physical	50.00 ± 28.58	69.56 ± 32.35	45.66 ± 27.74	70.54 ± 26.02
Bodily pain	57.18 ± 24.07	80.55 ± 20.59	55.19 ± 27.33	76.14 ± 24.53
General health	66.28 ± 20.00	72.09 ± 19.53	62.08 ± 22.63	67.25 ± 19.54
Vitality	55.61 ± 17.13	63.91 ± 16.98	56.30 ± 16.73	63.79 ± 12.93
Social functioning	76.10 ± 25.81	85.16 ± 20.44	77.43 ± 23.87	88.21 ± 17.13
Role emotional	64.14 ± 29.79	78.23 ± 26.15	64.35 ± 31.60	79.05 ± 25.44
Mental health	64.85 ± 15.85	69.81 ± 13.70	64.78 ± 16.39	69.70 ± 12.17

### Pain score and daily questionnaire activity score

**Fig. 5:**
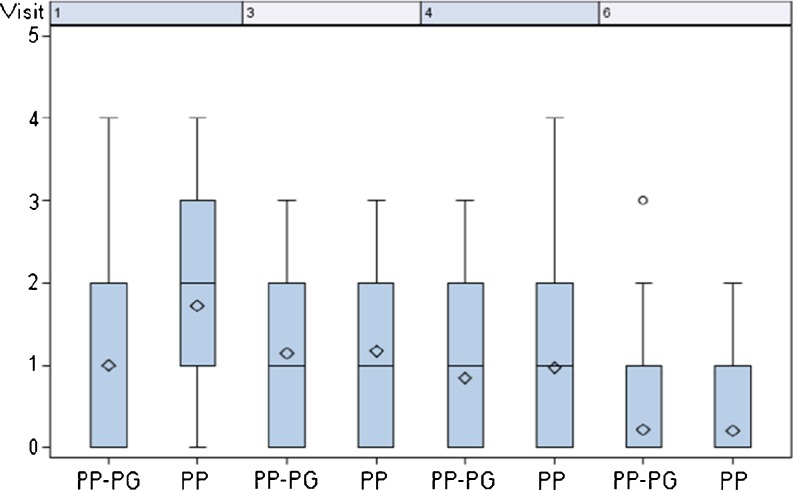
Pain score. Visit 1: pre-op; visit 3: day of discharge, visit 4: 21 days post-operatively, visit 6: 6 months post-operatively

**Fig. 6:**
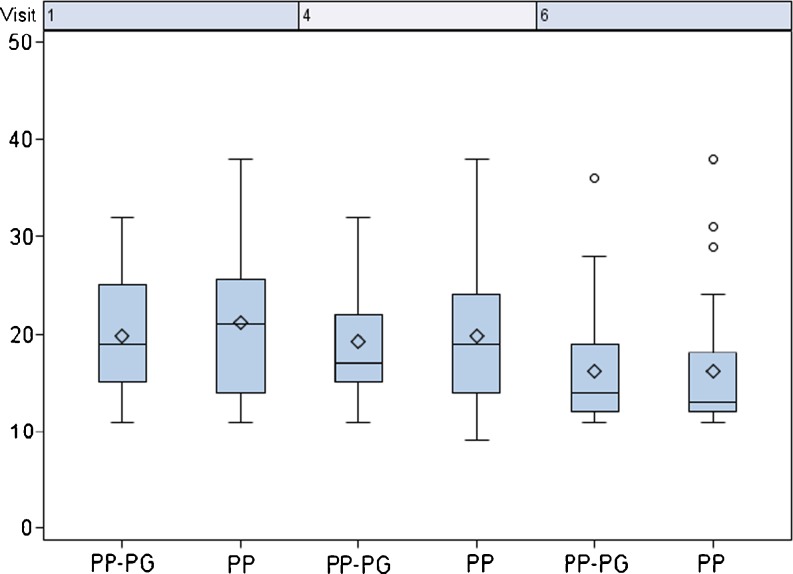
Daily activity score. Visit 1: pre-op; visit 4: 21 days post-operatively, visit 6: 6 months post-operatively

The measured pain score was similar in both groups at every visit (Fig. 5). The lowest pain score level was observed 6 months after operation (0.52 PP vs. 0.47 PP-PG). The pain score of patients with the non-absorbable PP mesh showed an improvement from pre-operative to post-operative (1.72 to 0.52), whilst patients with the PP-PG mesh showed a constant pain score over almost the whole observation period with a slight improvement towards the end of the observation (1.00 to 0.47). For the daily activity score, no significant difference was found (Fig. 6). Six months post-operatively, both groups showed an improvement compared to the pre-operative rating.

## Discussion

The standard procedure in incisional hernia repair is the implantation of a prosthetic mesh in sublay position resulting in recurrence rates between 2 and 12 % [8, 12, 16, 17]. This procedure is superior to direct suture techniques with recurrence rates of up to 60 % and mesh augmentation in onlay or inlay technique with recurrence rates between 20 and 40 % [4, 6, 9, 18, 19]. However, mesh repair, especially in sublay technique, is more complex and thus requires a higher surgical expertise. Moreover, studies have shown a higher rate of wound infections and mesh-related seromas [9, 20, 21].

A great variety of meshes which differ in material, textile structure, pore size, weight and degree of absorbable components are available. The implanted foreign material induces the building of scar tissue in the abdominal wall which together with a contraction of the mesh can lead to reduced abdominal wall flexibility resulting in chronic pain and limitations in daily activity [22–24]. Lightweight, large pore-sized meshes are used widely in mesh hernia repair. They seem to result in an optimal stability maintaining the physiological flexibility of the abdominal wall even after healing incompletely. Several studies found a better abdominal wall function and less chronic pain for lightweight meshes compared to the standard and heavyweight meshes [23, 37]. Nevertheless, prospective studies dealing with the question of which type of mesh to implant in which position are rare [10, 12, 25–29]. A recently updated review came to the conclusion that due to a lack of trials, no final conclusion in regard to this question could be drawn [9].

Only few prospective studies have investigated the impact of specific mesh materials on patients' quality of life. Mostly standard or heavyweight meshes were compared with lightweight meshes [11, 12]. No randomised controlled trial has hitherto evaluated if the absorbable part of a lightweight mesh influences complication rates, patients' discomfort and pain.

Experiments in animals showed no difference in tensile strength and rupture force between partly absorbable and non-absorbable meshes 3 months after implantation, though the partly absorbable meshes showed less adhesion formation on the peritoneum-facing surface [30–32]. Theoretically, less adhesion formation and foreign material that persists in the patient could lead to improved abdominal wall elasticity, in turn, resulting in a positive effect on patients' quality of life and pain. At least in the case of inguinal hernias, this could not be confirmed in clinical practice: all studies comparing partly absorbable with non-absorbable inguinal mesh hernia repair found no differences in pain score and quality of life [33, 34]. For incisional hernia, no studies investigating this are available.

In the presented prospectively randomised multicentre trial, we compared two lightweight meshes, the non-absorbable Optilene® Mesh Elastic and the partly absorbable Ultrapro® Mesh. The study focused on patient-related outcome, with different questionnaires measuring physical and mental health, daily activity and pain on defined follow-up dates. As the primary endpoint, the performance in the physical health score from the SF-36 questionnaire 21 days after mesh implantation was defined. Secondary variables included the evaluation of patients' daily activity and pain, as well as post-surgical complications and recurrence rates. No differences were observed for the SF-36 score, pain score and the daily activity questionnaire for both treatment groups after 21 days and 6 months, with a significant improvement from the pre-operative baseline to the 6-month post-operative measurement.

Since there are no other studies available comparing non-absorbable with partly absorbable lightweight meshes in incisional hernia repair, the data can only be discussed in the context of studies that deal with inguinal hernia repair. Here, a recent review analysing 3,133 inguinal hernia repairs reported an influence of mesh weight on pain and foreign body sensation, independent if a non-absorbable or a partly absorbable mesh was used [34]. A further study comparing an absorbable with a non-absorbable mesh in Lichtenstein hernioplasty also used the SF-36 questionnaire and found no differences in the SF-36 scores over a follow-up period of 1 year [35].

Only few studies have analysed quality of life after incisional hernia mesh repair. A recent study found no significant differences in SF-36 scores between the sublay implantation of a heavyweight vs. a partly absorbable, lightweight PP mesh [11]. The median follow-up in this study was 112 months, but the limited study population size of only 12 patients in each group severely limits the validity of this analysis. Conze et al. compared two non-absorbable PP heavyweight meshes and one polyester mesh with a partly absorbable lightweight mesh. Scores for the SF-36 and the daily activity questionnaire did not differ between the treatment groups [12]. Snyder et al. conducted a retrospective survey 5 years after elective incisional hernia repair. In total, 200 patients had received a mesh. They could not detect any effect of mesh texture on patient outcome (SF-36, pain score), but due to the retrospective character and the moderate methodological quality of this study, conclusions are difficult to draw (only 43 % of operated patients answered the survey, 23 % died between the operation and the survey, no detailed mesh characteristics are mentioned) [36].

Secondary endpoints in the present study were post-surgical complications and recurrence rates. In this study, no hernia recurrence was observed; this is probably mainly due to the restricted follow-up time of only 6 months as most hernia recurrences are known to usually occur later than 6 months after the operation [4].

Complication rates of mesh-related complications were similar for the two types of meshes used. Most complications could be managed without an operation. Minor haematomas and seromas were found in up to an quarter of patients but resolved in most patients within the follow-up period of 6 months without treatment. Wound/mesh infections could be treated conservatively in most cases. Infection rates in the literature range between 4 and 17 % [6, 21], which is well comparable with the given study. Only one partly absorbable mesh had to be removed because of severe mesh infection. Three patients needed surgery for major haematoma; this is also comparable to other studies [12]. With equal effectiveness, the implantation of a partly absorbable mesh leads to more material costs, as usually partly absorbable meshes have a higher market price than non-absorbable meshes; e.g., during the period of the trial in the German centres, the price for a partly absorbable mesh was approximately 1.3-fold higher.

A limitation of the study is that no sample size calculation was performed due to the lack of any empirical data for the primary endpoint in the population which was investigated. There was a high uncertainty regarding to the overall rate and treatment effects to be expected. On the other hand, a randomised, multi-centric study design with double blinding was performed. Furthermore, stringent inclusion and exclusion criteria and strictly standardised surgical procedures were used to ensure comparability of the study population. Therefore, the decision was made to include 80 patients, a number which could be recruited in six centres in an appropriate period of time. As this was the first randomised controlled trial analysing the quality of life in patients undergoing an incisional hernia repair, further randomised controlled trials should be conducted to confirm our results.

## Conclusion

In this study, the use of a partly absorbable vs. a non-absorbable mesh for incisional hernia repair did not influence the patients' quality of life as measured by the SF-36 score. Complication and recurrence rates did not differ between the two types of meshes. The results implicate that a partly absorbable mesh does not have any advantage in the improvement of the quality of life over a conventional non-absorbable lightweight PP mesh after incisional hernia repair.

## Abbreviations

AE: Adverse event
BMI: Body mass index
PG: Polyglecaprone
SF-36: Short Form 36
PP: Polypropylene
